# Exploring the effects of tinzaparin and cisplatin on lung cancer cells in vitro

**DOI:** 10.1186/s12935-026-04214-5

**Published:** 2026-02-05

**Authors:** Julia Held, Marc A. Schneider, Beatriz Martinez-Delgado, Bin Liu, David S. DeLuca, Elena Korenbaum, Sabina Janciauskiene, Thomas Muley

**Affiliations:** 1 Department of Respiratory Medicine, Biomedical Research in Endstage and Obstructive Lung Disease Hannover (BREATH), Member of the German Center for Lung Research (DZL), Hannover Medical School, Hannover, Germany; 2 Thoraxklinik,Translational Lung Research Center Heidelberg (TLRC), Member of the German Center for Lung Research (DZL), Heidelberg University Hospital, Heidelberg, Germany; 3 Department of Molecular Genetics, Center for Biomedical Research in the Network of Rare Diseases (CIBERER), Institute of Health Carlos III, Majadahonda, Spain; 4 Division of Structural Biochemistry, Hannover Medical School, Hannover, Germany

**Keywords:** Lung cancer, Low molecular weight heparin, Tinzaparin, Cisplatin, Cell viability, Cell proliferation, RNA sequencing

## Abstract

**Background:**

Low molecular weight heparins (LMWH) are widely used to prevent or treat cancer- and thrombosis-related conditions. However, their direct effects on cancer cells remain unclear.

**Methods:**

Nine non-small cell lung cancer (NSCLC) cell lines (H1437, H2126, H661, H1299, H1563, H1975, H1573, 2106 T, 2427 T) were treated with tinzaparin (50 IU/ml, ~ 76.9 µM), cisplatin (15 µg/ml), or a combination. We assessed cell viability, proliferation, colony formation, and intracellular lipid droplet accumulation. RNA sequencing was performed on two adenocarcinoma (H1437, H1563) and two squamous cell carcinoma (2106 T, 2427 T) lines to explore transcriptomic changes.

**Results:**

Tinzaparin increased proliferation in H1437, H2126, H1299, and H661 cells, but slightly reduced it in 2427 T cells. Colony formation was reduced only in 2427 T, with no effect in other lines despite some showing increased proliferation. Tinzaparin modestly increased viability in 2427 T cells and partially counteracted cisplatin-induced proliferation inhibition in H1573 and apoptosis in H1437 and H2126. Lipid droplet formation was unaffected. Transcriptomic analysis showed variable responses across cell lines, with only *CTSL* (Cathepsin L) and *HMOX1* (Heme Oxygenase 1) consistently altered by tinzaparin.

**Conclusion:**

Tinzaparin affects proliferation, viability, colony formation, and drug sensitivity in NSCLC cells in a cell type–specific manner. These findings highlight the complex actions of LMWHs on lung cancer cells and underscore the need for further mechanistic studies to understand their potential impact on cancer progression and therapy.

**Supplementary Information:**

The online version contains supplementary material available at 10.1186/s12935-026-04214-5.

## Background

Lung cancer is a leading cause of cancer incidence and mortality worldwide [1]. The two main types of lung cancer are small cell lung cancer (SCLC) and non-small cell lung cancer (NSCLC); the latter one comprising about 85% of all cases [2]. The most common subtypes of NSCLC are adenocarcinoma, squamous cell carcinoma, and large cell carcinoma [2]. Although NSCLC is strongly associated with cigarette smoking, adenocarcinomas also occur in never smokers, mostly associated with molecular alterations [3].

Compared to the general population, cancer patients show significantly higher risk of arterial and venous thromboembolic events [4, 5]. The prevalence of thrombotic complications in cancer patients depends on various factors, including tumour stage, location of metastases, body mass index as well as anti-cancer therapies [6–8]. For example, the use of anti-vascular endothelial growth factor drugs in advanced NSCLC might increase a risk of arterial thromboembolism [9]. Moreover, cancer cells per se can create an imbalance in the pro- and anti-coagulation system and thus, thrombotic processes in cancer patients may differ from non-cancer patients [10, 11]. Previous studies indicated that anticoagulants themselves, specifically low molecular weight heparins (LMWHs), might have a positive effect on cancer patient outcome. According to a small, prospective, randomized single-centre study, the administration of dalteparin significantly prolonged overall survival of SCLC patients with various disease stages [12]. Likewise, a multicentre, open label, randomized, controlled trial in patients with solid tumours and venous thrombosis showed that dalteparin improves patients’ survival [13]. Although the outcomes of the initial clinical studies on the anti-tumour effects of LWMH were intriguing, other studies gave conflicting results [14]. For example, a systematic review and meta-analysis of randomized trials conducted by Sanford and co-authors reported that the use of LMWH has no effect on mortality [15]. In line, a systematic review and meta-analysis of randomized controlled trials to determine the impact of primary ambulatory thromboprophylaxis with LMWHs on overall survival in patients with lung cancer showed no survival advantage [16]. In this context, the results of experimental studies suggesting that LMWH could affect the growth, adhesion, invasion and angiogenesis of cancer cells are also controversial [17–19].

Tinzaparin is a widely used LMWH-anticoagulant in various European countries, particularly for the management of cancer-associated thrombosis, including in patients with lung cancer [20]. Current research supports the hypothesis that LMWH, like tinzaparin, may also exert beneficial effects beyond anticoagulation by modulating cancer progression. In vitro studies have demonstrated both anti-oncogenic and anti-metastatic effects of tinzaparin [21]. Some researchers have proposed that tinzaparin can augment the effectiveness of chemotherapeutic drugs [22]. Encouraged by the recent findings, in this study we aimed to investigate the anti-cancer effects of tinzaparin alone and in combination with cisplatin on several lung cancer cell lines in vitro*.*

## Material and methods

### Cancer cell culture

The NSCLC cell lines NCI-H661 (ATCC® HTB-183™), NCI-H1299 (ATCC® CLR-5803™), NCI-H1437 (ATCC® CRL-587™), NCI-H1563 (ATCC® CRL-5875™), NCI-H1573 (ATCC® CRL-5877™), NCI-H1975 (ATCC® CRL-5908™) and NCI-H2126 (ATCC® CCL-256™) were purchased from ATCC (Virginia, MA, USA). The well-characterized cell lines 2106 T and 2427 T were a gift from Dr. Marc Schneider, Heidelberg [23]. All cell lines were cultured without antibiotics at 37 °C, 5% CO_2,_ using the appropriate growth media (Table 1).

**Table 1:** Main characteristics of the cell lines used in this study

Cell line	Tumour type	Source	Mutant genes	Complete medium
H1975	primary tumour	NSCLC, adenocarcinoma	CDKN2A, EGFR, PIK3SCA, TP53	RPMI-1640 + 10% FBS
H1563	primary tumour	NSCLC, adenocarcinoma	CDKN2A	RPMI-1640 + 10% FBS
H1437	metastasis, pleural effusion	NSCLC, adenocarcinoma	CDKN2A, TP53	RPMI-1640 + 10% FBS
H2126	metastasis, pleural effusion	NSCLC, adenocarcinoma	CDKN2A, STK11, TP53	DMEM/F-12 with HEPES and L-Glutamine + 5% FBS + 2 mM L-glutamine + 0.005 mg/ml insulin + 0.01 mg/ml apo-transferrin + 30 nM sodium selenite + 10 nM hydrocortisone + 10 nM beta-estradiol
H1573	metastasis, soft tissue	NSCLC, adenocarcinoma	KRAS, PIK3R1, SMARCA4, TP53	RPMI-1640 + 5% FBS
H661	metastasis, lymph node	NSCLC, large cell carcinoma	CDKN2A, SMARCA4, TP53	RPMI-1640 + 10% FBS
H1299	metastasis, lymph node	NSCLC, large cell carcinoma	p53 null, NRAS	RPMI-1640 + 10% FBS
2106 T	primary tumour	NSCLC, squamous cell carcinoma	CDKN2A	DMEM/F-12 + 10% FBS + 2 mM L-glutamine + 25 mM HEPES
2427 T	primary tumour	NSCLC, squamous cell carcinoma	TP53	DMEM/F-12 + 10% FBS + 2 mM L-glutamine + 25 mM HEPES

DMEM/F-12 with HEPES and L-Glutamine, DMEM/F-12 and RPMI-1640 medium were purchased from Life Technologies, Paisley, UK; fetal bovine serum (FBS) and HEPES were from Gibco/Life Technologies, Bleiswijk, The Netherlands; L-glutamine was from LIFELINE Cell Technology, Frederick, Maryland, US; insulin, apo-transferrin, sodium selenite, hydrocortisone and beta-estradiol were from Sigma Aldrich Chemie GmbH, Steinheim, Germany

### Drugs used for cancer cell treatment

The LMWH tinzaparin sodium was purchased from LEO Pharma A/S, Ballerup, Denmark. The therapeutic dosages of LMWH used in cancer patients are typically approximately 175 IU·kg − 1 once a day [21]. For the cancer cell treatment, a constant tinzaparin concentration of 50 IU/ml (corresponding to approximately 76.9 µM) was used. Cisplatin, a platinum-based chemotherapeutic, was purchased from Merck, Darmstadt, Germany and dissolved in 0.9% NaCl (Fresenius Kabi AG, Bad Homburg, Germany) at a concentration of 0.5 mg/ml. For experiments, cisplatin was used at a constant concentration of 15 µg/ml.

### Cell viability analysis using the PE Annexin V apoptosis detection kit

The cells were cultured in serum-free medium either alone, supplemented with tinzaparin, cisplatin or tinzaparin plus cisplatin for 48 h. Cell viability was assessed by flow cytometry (Luminex, Austin, Texas, USA) using the PE Annexin V Apoptosis Detection Kit (BD Pharmingen, San Diego, CA, USA) as described elsewhere [24]. Cells negative for Annexin V and 7-AAD were classified as “living”.

### Cell viability analysis using the XTT assay

Cells were seeded in 96-well plates and allowed to attach overnight. The following day, cells were left untreated or treated with tinzaparin, cisplatin, or a combination of both. Cell viability was assessed using the CyQUANT XTT Cell Viability Assay (Thermo Fisher Scientific, Waltham, MA, USA) following the manufacturer’s instructions. Absorbance was measured at 450 nm (XTT-specific signal) and 660 nm (background correction). Specific absorbance was calculated using the formula: Specific Absorbance (Abs) = (Abs 450 nm (Test) − Abs 450 nm (Blank)) − Abs 660 nm (Test).

### Cell proliferation analysis using the CyQUANT NF Cell Proliferation assay

The cells were seeded on 96-well plates at a density of 500 cells per well and allowed to attach for 18 h. Subsequently, the cells were treated with tinzaparin, cisplatin, or a combination of both in complete medium for 48 h at 37 °C in a 5% CO₂. Proliferation was then assessed using the CyQUANT NF Cell Proliferation Assay Kit (Thermo Fisher Scientific, Waltham, Massachusetts, USA) according to the manufacturer’s recommendations, which employs a cell-permeant DNA-binding dye and a plasma membrane permeabilization reagent. Fluorescence intensity was measured with a microplate reader (Infinite 200 PRO Microplate Reader, Tecan, Maennedorf, Switzerland), using an excitation wavelength of 485 nm and emission detection at 530 nm.

### Cell proliferation analysis using the bicinchoninic acid (BCA) assay

Cell lysates were prepared using RIPA buffer (50 mM Tris–HCl (pH 7.4), 150 mM NaCl, 1% NP-40 (Nonylphenoxypolyethoxylethanol), 0.5% Sodium deoxycholate, 0.1% SDS (Sodium dodecyl sulfate) supplemented with 0.1% protease inhibitor cocktail (Sigma-Aldrich, St. Louis, MO, USA). Protein concentrations were determined using the Pierce BCA Protein assay kit (ThermoScientific, Rockford, IL, USA) according to the manufacturer’s instructions. Optical density (OD) values were measured at a wavelength of 562 nm using the microplate reader Infinite M200 (Tecan, Männedorf, Switzerland). All measurements were performed in triplicates.

### Lipid droplet (LD) staining

The cells were cultured on coverslips in serum-free medium alone or with test substances for 48 h at 37 °C, 5% CO_2_. Following this, cells were fixed with 3% paraformaldehyde (Roth, Karlsruhe, Germany), treated briefly with 100% isopropanol, and stained with Oil Red O as previously described [25].

### Colony formation

The cells were seeded onto 6-well plates (H1299, H1563, H661, H1437, H2126, 2427 T: 500 cells per well; H1975, H1563, 2106 T: 1000 cells/well) and allowed to attach for 18 h at 37 °C in 5% CO₂. Subsequently, the cells were treated with tinzaparin for 24 h in serum-free medium. After treatment, the medium was replaced with the respective complete medium, and the cells were incubated for an additional 6 days at 37 ºC in 5% CO₂. We also performed an additional experiment using the H1437 cell line with continuous tinzaparin exposure. In this experiment, cells were plated in 6-well plates, allowed to attach overnight, and then treated with tinzaparin. The medium was replaced every 2 days with either complete medium alone (control) or complete medium supplemented with tinzaparin (treatment) for a total of 6 days. Post incubation, the cells were washed with phosphate buffered saline (PBS) and fixed with 100% methanol. For colony quantification, the cells were stained with 0.1% crystal violet, and images were captured with ChemiDoc Imaging System (Bio-Rad, Hercules, CA, USA). Cell colonies of predefined size were counted using Fiji [26].

### RNA sequencing and data analysis

The four cancer cells lines 2106 T, 2427 T, H1437 and H1563 were cultured in serum-free medium without or with tinzaparin for 48 h. RNA was isolated with the RNeasy Mini Kit (Qiagen, Hilden, Germany) according to the manufacturer’s instructions and RNA concentrations were measured using the Nanodrop 1000 Spectrophotometer (Peqlab Biotechnologie GmbH, Erlangen, Germany. RNA sequencing was done in triplicates as described previously [27]. Data normalization and differential expression analysis were performed on raw counts using the R package DESeq2 v1.32.0 based on default settings, namely Wald test for attaining p-values and the Benjamini and Hochberg method for multiple testing corrections [28, 29]. The normalized gene expression levels and DEG results were visualized using R and the related packages, including ggplot2 Version [3.3.5], ggrepel Version [0.9.1], and pheatmap Version [1.0.12] [30–32]. The data have been deposited in NCBI’s Gene Expression Omnibus and are accessible through GEO Series accession number GSE242413 (https://www.ncbi.nlm.nih.gov/geo/query/acc.cgi?acc=GSE242413).

### Statistical analysis

Data distribution was assessed using the Shapiro–Wilk test. For comparisons between two groups, normally distributed data were analyzed using an unpaired t-test, while non-normally distributed data were analyzed using the Mann–Whitney test. For comparisons among more than two groups, normally distributed data were analyzed using one-way ANOVA, and non-normally distributed data were analyzed using the Kruskal–Wallis test. Normally distributed data are presented as mean ± standard deviation (SD), whereas non-normally distributed data are presented as median with interquartile range (IQR). All analyses and visualizations were performed using GraphPad Prism 9 (GraphPad Software, San Diego, CA, USA). A p-value < 0.05 was considered statistically significant.

## Results

### Effect of tinzaparin and cisplatin on cancer cell proliferation

**Fig. 1:**
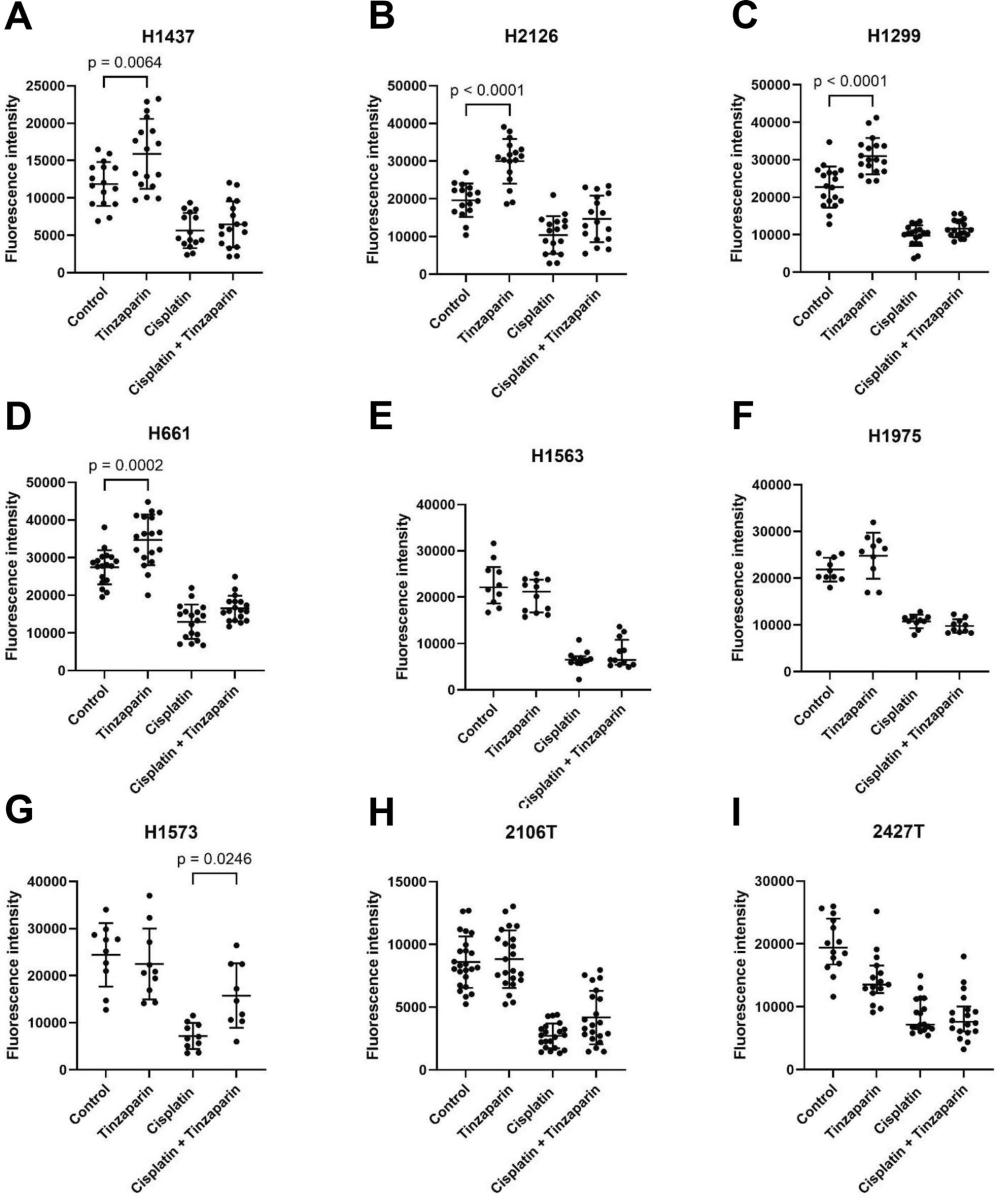
Effects of tinzaparin (LMWH) on cancer cell proliferation. Bars represent mean (SD). Cells were cultured in the respective complete medium alone or in the presence of tinzaparin or cisplatin, or both for 48 h. Cell proliferation was analysed by the NF CyQuant cell proliferation kit. Data were generated from 5 to 6 technical replicates from two or three independent experiments. (**A**-**D**; **F**–**H**): Data are normally distributed and shown as mean (SD); p-values were calculated using the One-way ANOVA test. (**E**, **I**): Data are not normally distributed and shown as median (IQR); p-values were calculated using the Kruskal–Wallis test. A p-value below 0.05 is considered as significant. P-values for non-significant comparisons of interest: control vs tinzaparin: H1563: p > 0.9999; H1975: p = 0.1273; H1573: p = 0.8987; 2106 T: p = 0.9769; 2427 T: p = 0.4267. Cisplatin vs cisplatin + tinzaparin: H1437: p = 0.8992; H2126: p = 0.1096; H1299: p = 0.5177; H661: p = 0.1556; H1563:p > 0.9999: H1975: p > 0,8834; 2106 T: p = 0.0782; 2427 T: p > 0.9999

Tinzaparin increased proliferation in H1437, H2126, H1299, and H661 cells, while slightly reduced proliferation in 2427 T cells (Fig. 1). To further confirm this effect, H1437 cells were analyzed for total protein content using a BCA assay, which revealed increased protein levels in tinzaparin-treated cells compared to untreated controls, supporting enhanced proliferation (Additional file 1). As expected, cisplatin reduced proliferation in all cell lines; however, tinzaparin partially attenuated cisplatin’s inhibitory effect in H1573 cells (Fig. 1).

### Effect of tinzaparin and cisplatin on cancer cell viability

**Fig. 2:**
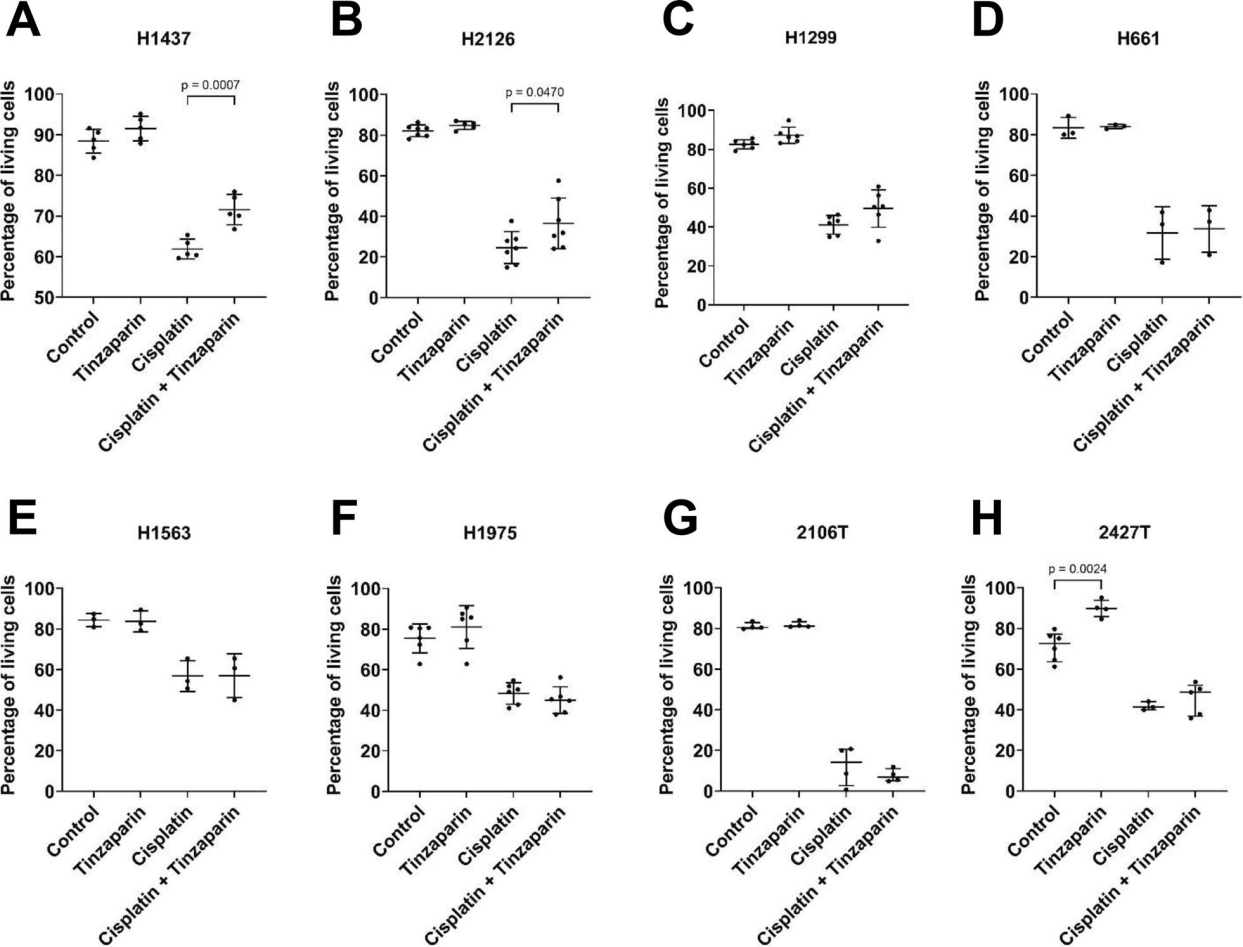
Tinzaparin and cisplatin effects on cancer cell viability. Cells were cultured in serum-free medium alone or in the presence of tinzaparin or cisplatin, or both for 48 h. Cell viability was analyzed by Annexin V Apoptosis Detection Kit and the percentage of living cells was determined (cells stained negative for both, PE Annexin V and 7-AAD). At least three independent experiments were performed for each cell line. (**A**-**G**): Data are normally distributed and presented as mean (SD); p-values were calculated using the One-way ANOVA test. (**H**): Data are not normally distributed and are shown as median (IQR); p-values were calculated using the Kruskal–Wallis test. A p-value below 0.05 was considered as significant. P-values for non-significant comparisons of interest: control vs tinzaparin: H1437: p = 0.4093; H2126: p = 0.9389; H1299: p = 0.5305; H661: p = 0.9997; H1563: p = 0.9994; H1975: p = 0.5888; 2106 T: > 0.9999. Cisplatin vs cisplatin + tinzaparin: H1299: p = 0.0974; H661: p = 0.9919; H1563: p > 0.9999; H1975: p = 0.8760; 2106 T: p > 0.9999; 2427 T: p = 0.8770

We next assessed whether tinzaparin influences cancer cell viability or modulates sensitivity to cisplatin. The H1573 cell line was excluded from flow cytometry analysis due to its tendency to form aggregates. Tinzaparin significantly increased viability in 2427 T cells but had no effect on the other cell lines. As expected, cisplatin markedly reduced viability in all cell lines; however, tinzaparin slightly attenuated cisplatin’s effect in H1437 and H2126 cells (Fig. 2A, B; Additional file 2).

### Effects of Tinzaparin on cancer cell colony formation

**Fig. 3:**
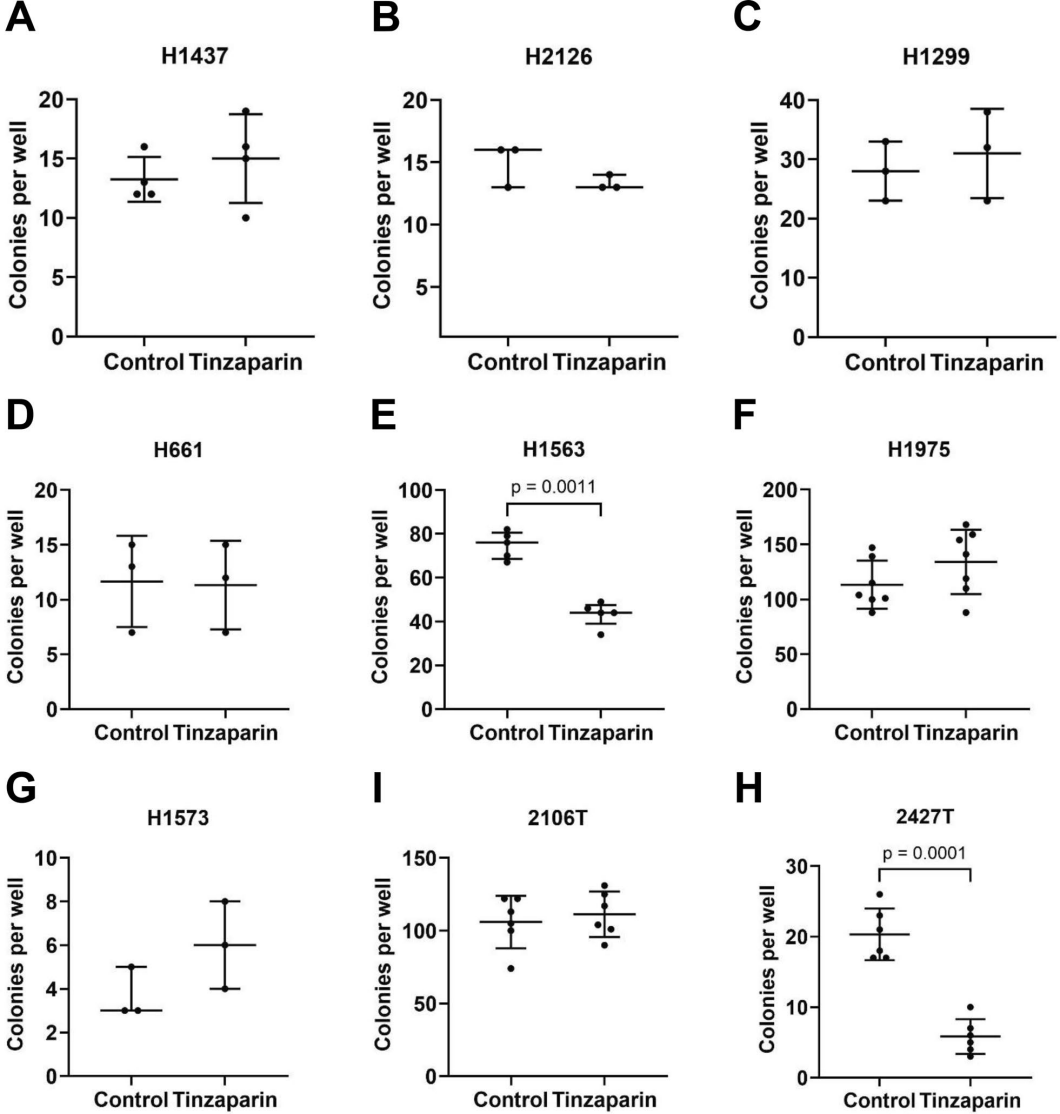
Effects of tinzaparin on cancer cell colony formation. Cells were treated with tinzaparin in serum-free medium for 24 h. The medium was then replaced with the respective complete medium, and the cells were further cultured for 6 days. Colonies were fixed, stained with crystal violet, and counted. (**A**, **C**-**F**, **H**-**I**): Data are normally distributed and shown as mean (SD); p-values were calculated using the unpaired t-test. (**B**, **G**): Data are not normally distributed and shown as median (IQR); p-values were calculated using the Mann–Whitney test. P-values for non-significant comparisons: H1437: p = 0.4359; H2126: p = 0.4000; H1299: p = 0.5968; H661: p = 0.9255; H1975: p = 1592; H1573: p = 0.2000; 2106 T: p = 0.5958

To further evaluate tinzaparin’s impact on cellular growth, colony formation assays were performed across multiple cell lines. Consistent with the proliferation data, 2427 T cells showed a significant reduction in colony number following tinzaparin treatment (Fig. 1I; 3I), and H1563 cells also exhibited decreased colony-forming capacity (Fig. 3E). In contrast, although H1437, H2126, H1299, and H661 cells displayed increased proliferation, their colony formation was unaffected. Similarly, no significant change was observed in 2106 T cells (Fig. 1A–D; Fig. 3A–D, H). To confirm these observations, we performed an additional experiment with H1437 cells using continuous tinzaparin exposure for six days, which similarly did not alter colony formation compared to control (Additional file 3). These results indicate that tinzaparin can enhance proliferation in certain cell lines without necessarily increasing their colony-forming ability in vitro.

### Effects of tinzaparin and cisplatin on intracellular lipid droplet (LD) accumulation

**Fig. 4:**
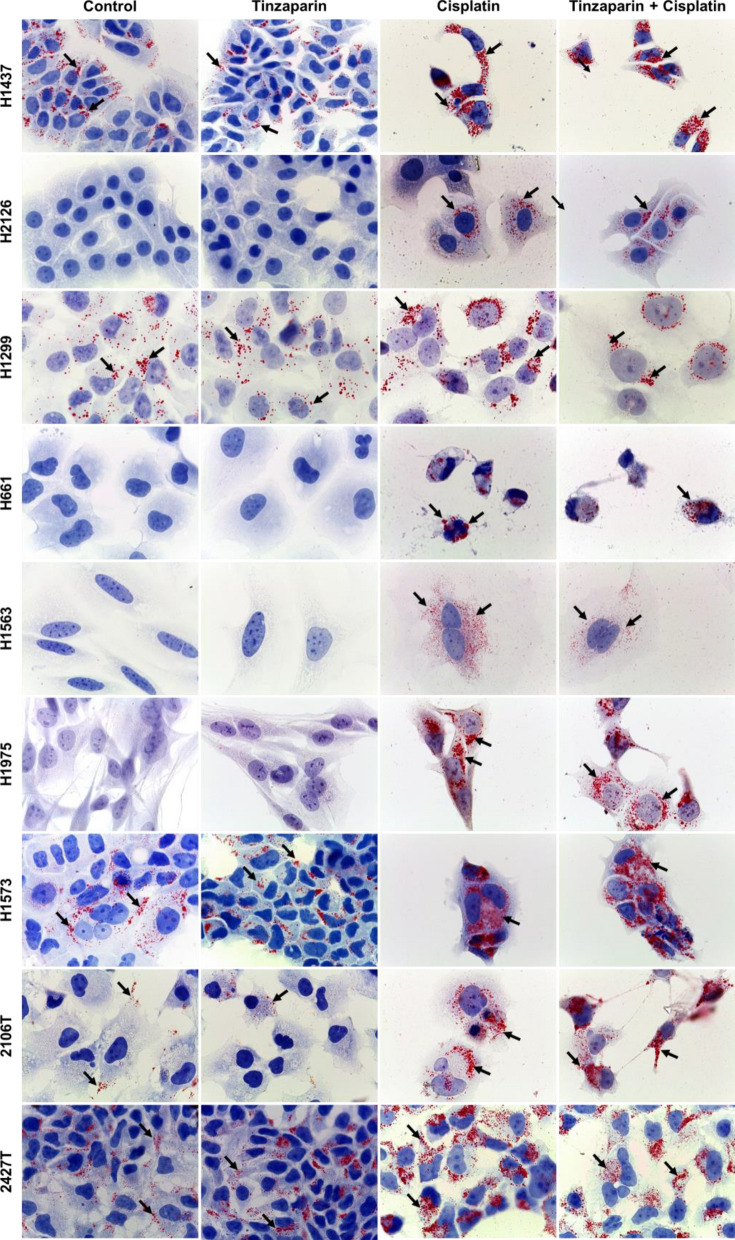
Effects of tinzaparin and cisplatin on lipid droplet (LD) formation. Cells were cultured alone or with tinzaparin or cisplatin separately or together for 48 h. Oil Red O Staining visualized the formation of LDs. Images were taken at 1000-fold magnification using a 100 × oil immersion objective. Images are representative out of four independent experiments. Black arrows indicate LDs

Lipid metabolism plays an important role in cancer, and LD accumulation is often associated with cancer cell proliferation and chemotherapy resistance [33]. As shown in Fig. 4, cisplatin induced LDs in H2126, H661, H1563 and H1975 cells, which are not forming LDs under basal conditions. Furthermore, cisplatin enhanced LD formation in 2106 T cells, which have low levels of LDs under basal conditions (Fig. 4). Tinzaparin itself had no effect on LD formation in any of the cell lines tested and had no significant influence on the effect of cisplatin (Fig. 4).

### Transcriptome analysis of four selected cancer cell lines

**Fig. 5:**
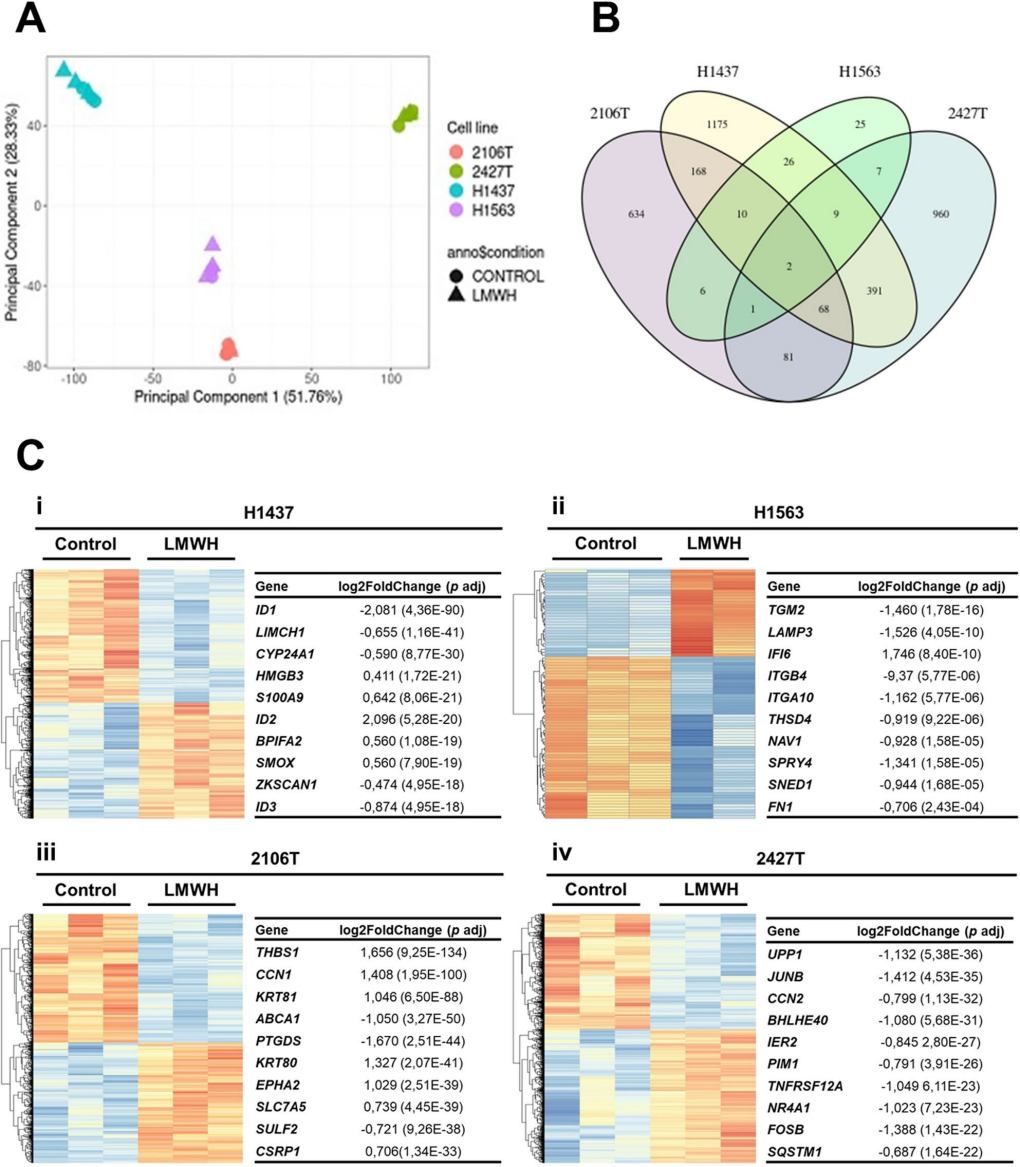
Effect of tinzaparin on the transcriptome of two adenocarcinoma cell lines H1437 and H1563 and two squamous cell carcinoma cell lines 2106 T and 2427 T. **A**: Principal component analysis (PCA). **B**: Overlap of differentially expressed genes (DEGs) in the tested cell lines. The Venn diagram illustrates the DEGs in tinzaparin-treated cells compared to non-treated control cells, with the highest number of DEGs observed in H1437 cells. **C**: Transcriptome analysis of cancer cell lines. Heat maps display gene expression in adenocarcinoma (H1437, **i**, and H1563, **ii**) and squamous cell carcinoma (2106 T, **iii**, and 2427 T, **iv**) cells. Normalized read counts were scaled row-wise at the gene level to enable comparability of expression levels across different genes (heatmap color: blue for low expression; red for high expression). The tables list the ten most significantly affected genes for each cell line, selected based on the lowest adjusted p-values (p adj)

To investigate the molecular effects of tinzaparin, we performed RNA sequencing on two adenocarcinoma cell lines (H1437 and H1563) and two squamous cell carcinoma cell lines (2106 T and 2427 T). Principal component analysis (PCA) showed that the four cell lines cluster distinctly based on their global gene expression profiles. Notably, the genes significantly regulated by tinzaparin varied considerably between the cell lines, highlighting cell line–specific transcriptomic responses (Fig. 5; Additional file 4). Specifically, we identified 970 differentially expressed genes (DEGs) in 2106 T cells (469 upregulated and 501 downregulated), 1519 DEGs in 2427 T cells (815 upregulated and 704 downregulated), 1849 DEGs in H1437 cells (860 upregulated and 989 downregulated), and 86 DEGs in H1563 cells (30 upregulated and 56 downregulated). Remarkably, only two genes, *CTSL* (Cathepsin L) and *HMOX1* (Heme Oxygenase 1, HO-1), were significantly affected by tinzaparin in all cell lines (Fig. 5 B). Both genes were downregulated in tinzaparin-treated 2106 T cells (with log2-transformed fold change values of -0.48 and -0.27, p < 0.001) and 2427 T cells (log2-transformed fold change values of -0.31 and -1.07, p = 0.02 and p < 0.001, respectively). On the other hand, these genes were upregulated in tinzaparin-treated H1437 (log2 transformed fold change values of 0.23 and 0.49, p < 0.001) and H1563 (log2 transformed fold change values of 0.58 and 0.9, p = 0.03, respectively) (Additional file 4).

To investigate the specific biological pathways affected by tinzaparin, we analysed the significant gene ontology (GO) terms retrieved through Gene Set Enrichment Analysis (GSEA) using Enrichr. The top ten most affected pathways for each cell line are highlighted in the (Additional file 5). Overall, our results revealed that few cancer cell-related pathways were significantly affected by tinzaparin in at least two of the cell lines, particularly those involved in apoptosis (GO:0042981, GO:0043069, GO:0043066, GO:2,001,234), and the cell cycle (GO:0007052, GO:0007346, GO:0031571, GO:1,901,990, GO:0051726, GO:0030330, GO:0006977, GO:1,901,992, GO:0000281). A complete list of the DEGs and the GSEA results can be found in Excel tables within Additional files 4 and 6.

## Discussion

Emerging evidence suggests that LMWHs, such as tinzaparin, may provide therapeutic benefits beyond their established anticoagulant effects, particularly in cancer. Recent studies indicate that tinzaparin can modulate tumor progression. For example, it has been shown to reduce the dissemination of human breast cancer cells to the lungs and inhibit lung tumor formation, supporting a potential antimetastatic role [34, 35]. However, contrasting findings have also been reported. Some studies found no significant effect of tinzaparin on cell proliferation in breast cancer models or on primary tumor growth in the B16F10 metastasis model [21, 36]. Conversely, other studies have described pro-tumorigenic effects of LMWHs, such as increased proliferation and migration in various colon cancer cell lines, as well as inhibition of apoptosis through upregulation of the anti-apoptotic protein Bcl-2 [37]. These divergent results highlight that the effects of LMWHs on cancer cells may be context- and cell type–dependent.

One relevant factor influencing therapeutic response in cancer is cisplatin resistance, a major limitation in the clinical management of many solid tumors. Cisplatin, a widely used platinum-based chemotherapeutic agent, exerts its anti-tumor effects primarily by forming DNA crosslinks, which trigger DNA damage responses, leading to cell cycle arrest and apoptosis. [38]. Resistance to cisplatin can arise through multiple mechanisms, including enhanced DNA repair, altered drug uptake or efflux, and evasion of apoptosis, all of which contribute to treatment failure and tumor progression [39]. Recent studies suggest that LMWHs may influence these cisplatin resistance mechanisms. For instance, in cisplatin-resistant A549/DDP lung cancer cells, LMWH treatment restored drug sensitivity by promoting proteasome-mediated degradation of the ABCG2 (ATP-binding cassette sub-family G member 2) efflux transporter, resulting in increased intracellular cisplatin accumulation and enhanced apoptosis [40]. Similarly, in cisplatin-resistant ovarian carcinoma A2780cis cells, tinzaparin was shown to reverse resistance by downregulating TCF (T-cell factor) and LEF (lymphoid enhancer-binding factor), transcription factors that mediate canonical Wnt/β-catenin signaling [41]. Furthermore, tinzaparin has been reported to act synergistically with cisplatin in oral squamous cell carcinoma cell lines, reinforcing the notion that it can enhance chemotherapeutic efficacy in specific contexts [42, 43]. Together, these findings support the hypothesis that tinzaparin may exert pleiotropic effects on cancer cells, influencing proliferation, survival, drug sensitivity, and potentially impacting other hallmarks of cancer, including migration and invasion.

Therefore, in this study, we selected tinzaparin to investigate its effects on proliferation, viability, and colony formation in NSCLC cell lines. To evaluate these effects, we established an experimental model in which cancer cells were cultured under serum-free conditions, either alone or in the presence of tinzaparin, cisplatin, or a combination of both. This model allowed us to assess cell viability, colony formation, and, in selected cell lines, transcriptional changes. We hypothesized that serum-free conditions provide a more accurate assessment of the direct effects of tinzaparin and cisplatin on cancer cells, minimizing the potential confounding influence of serum proteins that could mask or alter drug activity. For proliferation assays, however, cells were cultured in complete medium with serum to support optimal growth, ensuring that the experimental conditions reflected the physiological environment necessary for cellular expansion.

Overall, our findings support the hypothesis that tinzaparin exerts multifaceted effects on cancer cell proliferation, viability, and colony formation. Importantly, these effects are highly cell line–specific and do not consistently correlate across different NSCLC models. See Table 2, which summarizes the observed effects of tinzaparin on proliferation, viability, and colony formation across different NSCLC cell lines.

**Table 2:** Summary of tinzaparin’s effects on the behavior of NSCLC cell lines

Cell line	Proliferation	Viability	Resistance to cisplatin	Colony formation
H1437	**Increased**	Not affected	**Increased**	Not affected
H2126	**Increased**	Not affected	**Increased**	Not affected
H1299	**Increased**	Not affected	Not affected	Not affected
H661	**Increased**	Not affected	Not affected	Not affected
H1563	Not affected	Not affected	Not affected	**Reduced**
H1975	Not affected	Not affected	Not affected	Not affected
H1573	Not affected	Not analyzed	Not analyzed	Not affected
2106 T	Not affected	Not affected	Not affected	Not affected
2427 T	Not affected	**Increased**	Not affected	**Reduced**

For example, tinzaparin significantly enhanced proliferation in the metastatic adenocarcinoma cell lines H1437 and H2126, as well as in the large cell carcinoma lines H1299 and H661. In contrast, it slightly reduced proliferation in the squamous cell carcinoma line 2427 T and had no significant effect on the remaining cell lines. The effects of tinzaparin on proliferation did not always correspond to its effects on cell viability, suggesting that distinct signalling pathways may be involved. Other studies reported that tinzaparin can activate pathways related to stress response or growth factor signalling, which promote cell survival without necessarily driving cell cycle progression [44, 45]. Zhang et al. demonstrated that LMWHs, including tinzaparin, reduce oxidative stress and inhibit apoptosis in lung cancer cells via activation of the PI3K/Akt pathway, which could explain how tinzaparin enhances cell survival without directly stimulating cell proliferation [46].

We also observed that tinzaparin partially attenuated the anti-proliferative effects of cisplatin in H1573 cells, suggesting that it may counteract cisplatin’s activity in certain contexts. In H1437 and H2126 cells, tinzaparin reduced cisplatin-induced cell death, further supporting the notion that it can modulate cellular responses to chemotherapy. This effect may involve activation of pro-survival pathways like NF-κB, which can promote chemoresistance by increasing anti-apoptotic proteins and reducing cell death, often without changing proliferation [47, 48]. These findings suggest that tinzaparin may protect certain NSCLC cells from chemotherapy-induced death, contributing to chemoresistance even without directly affecting proliferation or the cell cycle.

Colony formation assays are used to measure cancer cells’ ability to proliferate and form tumors, with larger or more numerous colonies generally indicating higher tumorigenic potential [49, 50]. Tinzaparin reduced colony formation in 2427 T and H1563 cells but had little effect in H1437, H2126, H1299, H661, or 2106 T, even in lines with increased proliferation. This suggests that tinzaparin may affect cell adhesion and aggregation—key factors for colony formation—rather than directly altering proliferation. Supporting this, previous studies have shown that changes in cell adhesion can disrupt the cell clustering needed for colonies [51].

Lipid droplets (LDs) are dynamic organelles that store triglycerides and cholesterol esters, serving as energy reserves during stress or high metabolic demand. In cancer cells, LDs support survival, growth, and proliferation, and can influence responses to anti-cancer drugs [52]. For example, cisplatin can induce LD formation in some lung cancer cells, which is linked to cell death [25, 53]. In our study, tinzaparin, alone or with cisplatin, did not alter LD formation. This suggests that tinzaparin’s effects are independent of lipid metabolism and LD dynamics, likely acting through other pathways.

Together, our findings show the pleiotropic and cell line–specific effects of tinzaparin on proliferation, viability, colony formation, and chemoresistance. These latter prompted us to investigate how tinzaparin might modulate the cancer cell transcriptome, providing deeper insights into the genes and molecular pathways underlying the observed phenotypic changes. To this end, we selected two adenocarcinoma cell lines (H1437 and H1563) and two squamous cell carcinoma cell lines (2106 T and 2427 T), which displayed distinct responses to tinzaparin. Some lines exhibited potentially pro-tumorigenic effects, such as increased proliferation and reduced cisplatin sensitivity in H1437 cells, whereas others showed anti-tumorigenic responses, including decreased colony formation in 2427 T cells. By comparing these divergent cells, we aimed to identify the genetic and molecular pathways that mediate tinzaparin’s context-dependent effects.

As predicted, we observed significant differences in the transcriptomes of each cell line, indicating that lung cancer cells, regardless of subtype, adenocarcinoma or squamous cell carcinoma, exhibit unique molecular signatures that drive their behaviour. Key differences included variations in gene expression related to pathways involved in cell proliferation, apoptosis, DNA repair, cell cycle regulation, and metabolism. These variations likely account for the differing results observed in the functional assays when the cells were treated with tinzaparin.

Remarkably, tinzaparin significantly affected only two common genes, *HMOX1* and *CTSL*, across all four cell lines. Both genes were downregulated in tinzaparin-treated 2106 T and 2427 T cells and upregulated in tinzaparin-treated H1437 and H1563 cells. Cathepsin L (*CTSL*), a lysosomal acid cysteine protease, plays a role in tumour metastasis and chemotherapy resistance [54]. Similarly, higher expression of *HMOX1* (heme oxygenase-1) in cancer cells promotes proliferation and survival [55]. In general, increased expression of *HMOX1* and *CTSL* is linked to more aggressive cancer phenotypes, including enhanced tumor survival, chemotherapy resistance, and increased invasion and metastasis. Conversely, reduced expression of *HMOX1* and *CTSL* may indicate greater vulnerability to oxidative stress and treatments.

Further, GSEA identified significant alterations in pathways associated with apoptosis (e.g., GO:0042981, GO:0043069, GO:0043066) and cell cycle regulation (e.g., GO:0007052, GO:0007346, GO:0031571), providing mechanistic insights into how tinzaparin may influence proliferation, survival, and chemoresistance. These transcriptomic data offer a valuable framework for future studies aimed at experimentally validating the key genes and pathways through which tinzaparin modulates cancer cell behavior.

## Conclusions

Our study demonstrates that lung cancer cells respond differently to tinzaparin and its combination with cisplatin, likely due to variations in their molecular and genetic profiles. In some cell lines, including certain adenocarcinoma and large cell carcinoma subtypes (H1437, H2126, H1299, H661), tinzaparin enhanced proliferation and reduced cisplatin sensitivity. In contrast, other lines, such as H1563 and 2427 T, showed decreased colony formation in response to tinzaparin. Transcriptomic analysis revealed that tinzaparin differentially regulates key pathways involved in apoptosis, autophagy, and the cell cycle, providing a potential explanation for these cell line–specific effects. Understanding these differences may guide the development of more effective strategies that combine tinzaparin with chemotherapy to optimize therapeutic outcomes in lung cancer.

## Supplementary Information

Additional file 1.

Additional file 2.

Additional file 3.

Additional file 4.

Additional file 5.

Additional file 6.

## Data Availability

Research data supporting this publication are available from the corresponding author upon reasonable request.

## Abbreviations

BCA: Bicinchoninic acid
CTSL: Cathepsin L
GO BP: Gene ontology biological process
GO: Gene ontology
GSEA: Gene set enrichment analysis
HMOX1/HO-1: Heme oxygenase-1
LD: Lipid droplet
LMWH: Low molecular weight heparin
NSCLC: Non-small cell lung cancer
PCA: Principal component analysis
SCLC: Small cell lung cancer
