# Supplementary material for: Exploring the effects of tinzaparin and cisplatin on lung cancer cells in vitro
Source: Cancer Cell Int. 2026 Feb 5;26:106. doi: 10.1186/s12935-026-04214-5 (PMC12934117; doi:10.1186/s12935-026-04214-5)
Supplement: Supplementary file 1 — Additional file 1. [file 12935_2026_4214_MOESM1_ESM.docx]

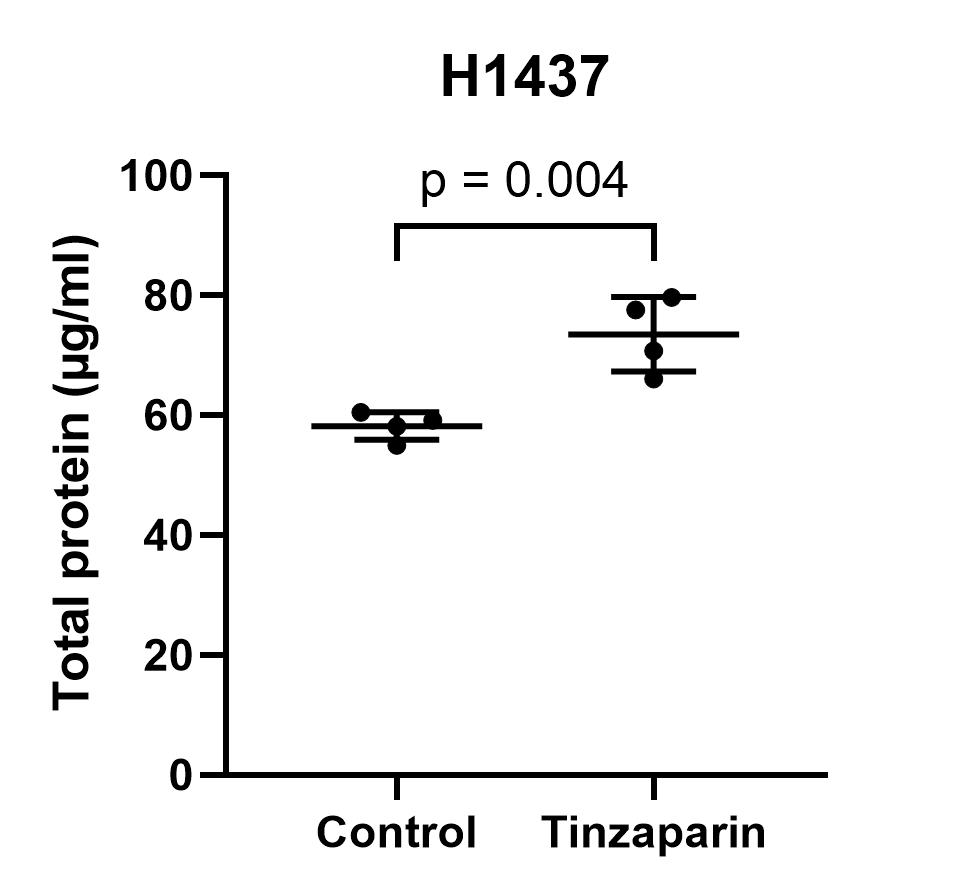


**Protein determination in H1437 cells upon treatment with tinzaparin.** H1437 cells were left untreated or were treated with tinzaparin for 48 h. Following, the total protein concentration was determined using the BCA assay. The normally distributed data were statistically analyzed using an unpaired t-test; a p-value < 0.05 was considered significant.
