# Supplementary material for: Exploring the effects of tinzaparin and cisplatin on lung cancer cells in vitro
Source: Cancer Cell Int. 2026 Feb 5;26:106. doi: 10.1186/s12935-026-04214-5 (PMC12934117; doi:10.1186/s12935-026-04214-5)
Supplement: Supplementary file 2 — Additional file 2. [file 12935_2026_4214_MOESM2_ESM.docx]

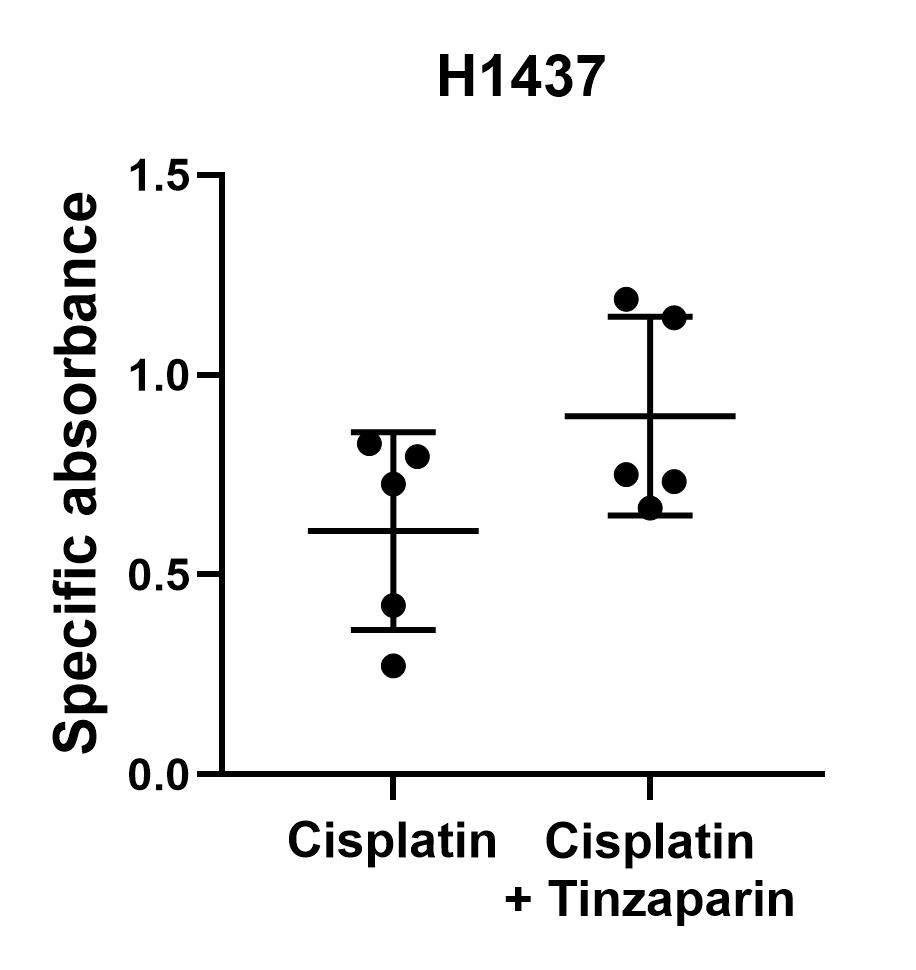


**Analysis of cell viability using the XTT cell viability assay.** H1437 cells were treated with cisplatin alone or in combination with tinzaparin for 48 h in basal medium. Following, cell viability was assessed using the XTT cell viability assay. Normally distributed data were statistically analysed using the One-way ANOVA test; a p-value < 0.05 was considered significant. P-value cisplatin vs cisplatin + tinzaparin: 0.104.
