# Supplementary material for: Exploring the effects of tinzaparin and cisplatin on lung cancer cells in vitro
Source: Cancer Cell Int. 2026 Feb 5;26:106. doi: 10.1186/s12935-026-04214-5 (PMC12934117; doi:10.1186/s12935-026-04214-5)
Supplement: Supplementary file 3 — Additional file 3. [file 12935_2026_4214_MOESM3_ESM.docx]

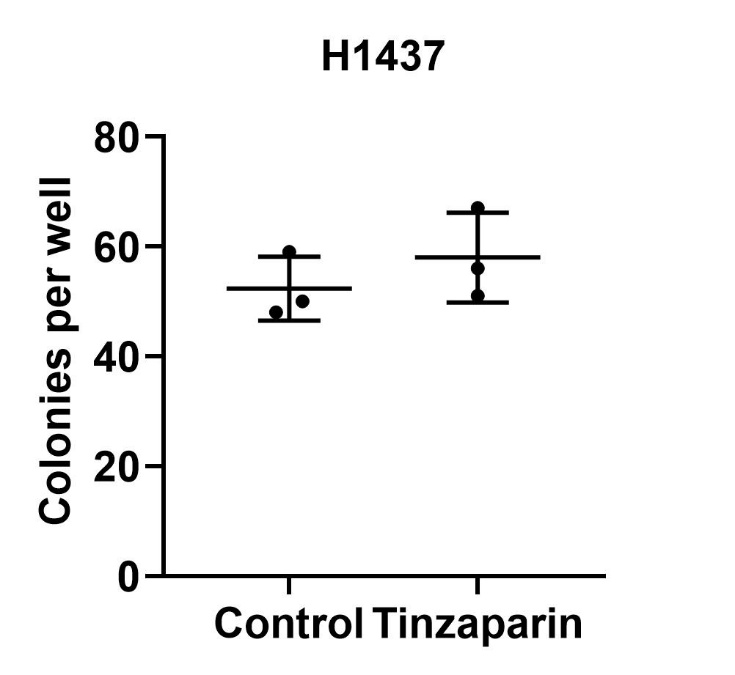


Figure 1: **Colony formation assay with continuous treatment with tinzaparin. Cells were seeded on 6 well plates and allowed to attach overnight. The next day, the cells were left untreated or treated with tinzaparin. The medium was replaced every 2 days with either complete medium alone (control) or complete medium supplemented with tinzaparin (treatment) for a total of 6 days.** The figure shows one experiment with three repeats; the normally distributed data were analyzed using an unpaired t-test; P-value control vs tinzaparin =0.3848.
