# Supplementary material for: Exploring the effects of tinzaparin and cisplatin on lung cancer cells in vitro
Source: Cancer Cell Int. 2026 Feb 5;26:106. doi: 10.1186/s12935-026-04214-5 (PMC12934117; doi:10.1186/s12935-026-04214-5)
Supplement: Supplementary file 5 — Additional file 5. [file 12935_2026_4214_MOESM5_ESM.docx]

**Additional data file 2. Top ten affected pathways per cell line.**

| ***Cell line: H1437*** | | |
| --- | --- | --- |
| **Term** | **Affected genes/Total number of genes** | ***p* adj** |
| GO:0006613: Cotranslational protein targeting to membrane | 54/94 | 4.81E-28 |
| GO:0006614: SRP-dependent cotranslational protein targeting to membrane | 51/90 | 2.93E-26 |
| GO:0045047: Protein targeting to ER | 54/103 | 8.52E-26 |
| GO:0002181: Cytoplasmic translation | 51/93 | 1.25E-25 |
| GO:0000184: Nuclear-transcribed mRNA catabolic process, nonsense-mediated decay | 52/113 | 1.60E-21 |
| GO:0000956: Nuclear-transcribed mRNA catabolic process | 64/171 | 7.78E-21 |
| GO:0043043: Peptide biosynthetic process | 61/162 | 5.16E-20 |
| GO:0006412: Translation | 68/214 | 9.53E-18 |
| GO:0044267: Cellular protein metabolic process | 99/417 | 3.63E-16 |
| GO:0010467: Gene expression | 89/356 | 5.88E-16 |
| ***Cell line: H1563*** |  |  |
| **Term** | **Affected genes/Total number of genes** | ***p* adj** |
| GO:0019646: Aerobic electron transport chain | 9/70 | 9.52E-09 |
| GO:0042775: Mitochondrial ATP synthesis coupled electron transport | 9/71 | 9.52E-09 |
| GO:0042776: Mitochondrial ATP synthesis coupled proton transport | 6/17 | 1.48E-08 |
| GO:0006123: Mitochondrial electron transport, cytochrome c to oxygen | 6/17 | 1.48E-08 |
| GO:0015986: ATP synthesis coupled proton transport | 6/19 | 2.57E-08 |
| GO:0042407: Cristae formation | 6/28 | 2.89E-07 |
| GO:0007007: Inner mitochondrial membrane organization | 6/49 | 8.54E-06 |
| GO:0043536: Positive regulation of blood vessel endothelial cell migration | 4/48 | 6.13E-03 |
| GO:0006868: Glutamine transport | 2/7 | 3.76E-02 |
| GO:0046580: Negative regulation of Ras protein signal transduction | 3/35 | 3.97E-02 |
| ***Cell line: 2106T*** |  |  |
| **Term** | **Affected genes/Total number of genes** | ***p* adj** |
| GO:0030334: Regulation of cell migration | 66/408 | 1.88E-14 |
| GO:2000147: Positive regulation of cell motility | 44/221 | 1.76E-12 |
| GO:0019221: Cytokine-mediated signaling pathway | 79/621 | 3.86E-12 |
| GO:0030335: Positive regulation of cell migration | 48/269 | 3.86E-12 |
| GO:0030198: Extracellular matrix organization | 46/300 | 3.28E-09 |
| GO:0042127: Regulation of cell population proliferation | 82/764 | 7.44E-09 |
| GO:0042981: Regulation of apoptotic process | 79/742 | 2.31E-08 |
| GO:0048522: Positive regulation of cellular process | 66/625 | 1.22E-06 |
| GO:0051091: Positive regulation of DNA-binding transcription factor activity | 36/246 | 1.46E-06 |
| GO:0043062: Extracellular structure organization | 33/216 | 1.98E-06 |
| ***Cell line: 2427T*** |  |  |
| **Term** | **Affected genes/Total number of genes** | ***p* adj** |
| GO:0006613: Cotranslational protein targeting to membrane | 37/94 | 3.00E-14 |
| GO:0006364: rRNA processing | 50/173 | 1.02E-13 |
| GO:0016072: rRNA metabolic process | 48/162 | 1.02E-13 |
| GO:0002181: Cytoplasmic translation | 35/93 | 3.08E-13 |
| GO:0006614: SRP-dependent cotranslational protein targeting to membrane | 34/90 | 5.72E-13 |
| GO:0042254: Ribosome biogenesis | 51/192 | 8.33E-13 |
| GO:0045047: Protein targeting to ER | 36/103 | 1.02E-12 |
| GO:0000184: Nuclear-transcribed mRNA catabolic process, nonsense-mediated decay | 37/113 | 3.97E-12 |
| GO:0000956: Nuclear-transcribed mRNA catabolic process | 46/171 | 8.35E-12 |
| GO:0034470: ncRNA processing | 50/201 | 1.60E-11 |

ECOG = Eastern Cooperative Oncology Group, NSCLC = non-small cell lung cancer, SCLC = small cell lung cancer.
